# Patterns of Pain and Functional Improvement in Patients with Bone Metastases after Conventional External Beam Radiotherapy and a Telephone Validation Study

**DOI:** 10.1155/2011/601720

**Published:** 2011-01-17

**Authors:** Liang Zeng, Arjun Sahgal, Liying Zhang, Kaitlin Koo, Lori Holden, Florencia Jon, May Tsao, Elizabeth Barnes, Cyril Danjoux, Kristopher Dennis, Luluel Khan, Edward Chow

**Affiliations:** Rapid Response Radiotherapy Program, Department of Radiation Oncology, Odette Cancer Centre, Sunnybrook Health Sciences Centre, University of Toronto, 2075 Bayview Avenue, Toronto, ON, Canada M4N 3M5

## Abstract

Patients experiencing lower body pain resulting from bone metastases have greater levels of functional interference than those with upper body pain. The purpose of this study was to assess the levels of interference caused by pain after treatment with conventional radiotherapy using the Brief Pain Inventory (BPI) and to validate this tool for telephone use. After radiotherapy, a total of 159, 129, and 106 patients completed the BPI over the telephone at months 1, 2, and 3, respectively. Cronbach's alpha, confirmatory factor analysis, and discriminant validity tests were performed to assess the validity of the BPI. One-way ANOVA was used to compare BPI scores. There was no statistically significant difference in functional interference among patients after treatment. Internal consistency of the BPI was high. Functional interference may be inherently higher in patients with pain in the lower body. Telephone use of the BPI is reliable and recommended in this population.

## 1. Introduction

Bone metastases can occur in up to 70% of patients with advanced cancer [1], and the resulting pain can lead to an overall decrease in quality of life [2–5]. Conventional external beam radiotherapy is a common treatment modality for bone metastases, and its efficacy is well established [3]. Other treatments can include analgesic therapy, orthopedic interventions (such as minimally invasive procedures and surgery), radionuclides, systemic therapies, and stereotactic body radiotherapy [6]. 

Bone metastases have received much attention in the literature regarding effective treatments and management of pain resulting from the disease [1, 7–9]. The characterization of other functional aspects such as pain interference (that with walking, sleep, and work) has been reported less though. Since the goal of treatment for bone metastases is mostly palliative, functional interference is an important factor which must be considered when assessing the quality of life (QoL) for these patients. 

The Brief Pain Inventory (BPI), developed by Cleeland and Ryan [10], has been validated for use in advanced cancer patients to assess pain and functional interference stemming from bone metastases [11]. It was reported that prior to treatment, patients with lower body bone metastases experience greater levels of functional interference than those with upper body metastases using the BPI [11]. The characterization of functional interference in patients with painful bone metastases is important for the management and treatment of these patients and could help especially with the prioritization of treatments. The purpose of this study was to report the patterns of pain and interference after treatment with conventional external beam radiotherapy, and determine if upper and lower skeletal index sites continue to demonstrate different interference levels.

The BPI is usually administered to patients prior to treatment in person. When following palliative patients, it is beneficial to reduce their number of visits to a health care centre. Studies in the past [12–15] have usually collected follow-up data across the telephone although this strategy has not been validated. Therefore, our secondary objective was to validate the BPI in patients with bone metastases receiving conventional radiotherapy using the telephone follow-up method.

## 2. Methods

### 2.1. Demographics

The Rapid Response Radiotherapy Program (RRRP) at the Odette Cancer Centre, Toronto, Canada provides rapid access to palliative radiotherapy. During the period lasting from May 2003 to June 2007, patients with bone metastases referred to the RRRP and subsequently treated with palliative radiotherapy were screened for eligibility for this prospective study assessing pain and functional interference using the Brief Pain Inventory (BPI). The institution's research ethics board had approved this study prior to commencement. Baseline data was collected, and patients were followed at four-week intervals following the end of radiotherapy for a total of 12 weeks (three followups in total). Patients were contacted by telephone for these followups by a trained research assistant. The BPI questions were read out loud, and the patient did not have a physical copy present when answering the items.

### 2.2. The Brief Pain Inventory (BPI)

The BPI is a validated multidimensional pain assessment tool developed by Cleeland and Ryan [10] which is often used to assess pain caused by bone metastases. It has been translated into many languages and satisfies two recommendations (assessment of pain to include both intensity and interference) set by the Initiative on Methods, Measurements, and Pain Assessment in Clinical Trials (IMMPACT) group [16]. Recently, research has suggested that a three-factor analysis (pain, affect interference, and activity interference) could yield stronger results while satisfying an additional IMMPACT recommendation [11].

Three questions regarding pain intensity and seven regarding pain interference are rated on an ordinal numerical scale with anchors of 0 (no pain/interference) to 10 (maximum pain/interference). Pain intensity is measured according to the worst pain experienced in the last three days, average pain in the last three days, and current pain. Pain interference assesses how that pain has affected general activity, mood, walking ability, normal work, relations with others, sleep, and enjoyment of life.

### 2.3. Statistical Analysis

Demographic results were expressed as means, standard deviations (SD), medians and interquartiles for continuous variables, and proportion for categorical values. Patients with complete data (no missing items on the BPI) were used for analysis. All analyses were repeated at all three follow-up intervals where data was collected. All analyses (one-, two-, and three-factor) were used to validate the psychometric properties of the BPI.

### 2.4. Analysis of Upper and Lower Skeletal Pain

Patients were separated into groups based on the location of pain: upper or lower skeletal metastases. Upper pain included patients receiving treatment to cervical or thoracic spine, shoulder girdle/upper extremity, ribs, and skull. Those receiving treatment to the lumbar spine, sacrum, or any of the pelvic girdle (iliac wing, acetabulum, pubic bone, ischial tuberosity), femur (head, neck, or shaft), and tibia were classified as having “lower skeletal pain”. Those receiving treatment to the thoracolumbar spine were excluded from this portion of the analysis to prevent inaccurate reporting of pain location. One-way ANOVA was applied for comparing mean scores of BPI subscales in patients with lower or with upper skeletal bone metastases at different follow-ups and also at baseline.

### 2.5. Item Analysis and Internal Consistency

With two- and three-factor analysis, item-item correlations were examined to identify redundant questions. Standardized Cronbach's alpha was applied to estimate internal consistency within each subscale [17]. Changes in the Cronbach's alpha were determined by removing individual items. Decreases in alpha after removal mean strong correlation with other items whereas increases describe weak correlation meaning the removal makes the construct more reliable.

### 2.6. Confirmatory Factor Analysis (CFA)

CFA was used to examine the structure of the BPI as a single construct (one-factor, null 10-item BPI model, with all items included), two-factor (pain and interference), and three-factor model (pain, affect, activity) for each follow-up period (4, 8, and 12 weeks) [18]. Covariance terms for the two-factor and three-factor models were generated and repeated with the removal of the sleep item [11]. Models were compared using various model-fit statistics [19] including (1) adjusted goodness of fit index (perfect fit = 1), (2) Chi-square statistic, which represents the value of the statistical criterion minimized in maximum likelihood estimation (smaller value = better fit), (3) root mean square error of approximation (RMSEA) with 90% upper level of confidence intervals, measuring the lack of fit of the model to the population covariance matrix (RMSEA ≥ 0.10 suggests poor fit), (4) Bentler's comparative fit index (CFI) and nonnormed fit index (NNFI), which measures the improvement in the overall fit and model complexity (above 0.9 suggests acceptable model fit).

Standardized factor loadings, associated statistics (i.e., *R*-squared and *t*-statistic), and composite reliability were provided for the best two-factor and three-factor models. Composite reliability is a measure of the overall reliability of a collection of heterogeneous but similar items. The minimum acceptance level of composite reliability is 0.70, and the minimum critical *t* value is 3.29 for *P* = .001. 

Discriminant validity tests (Chi-square difference test, confidence interval test, and variance extracted test) were carried out to further evaluate highly correlated factors within the three-factor model. The variance extracted estimates the amount of variance that is explained by an underlying factor in relation to the amount of variance due to measurement error. Fornell and Larcker suggested that constructs should exhibit estimates of 0.50 or larger [20]. It should be noted that Hatcher [18] cautions that the variance-extracted estimate test is conservative; reliabilities can be acceptable even if variance-extracted estimates are less than 0.50.

All analyses were performed using Statistical Analysis System (SAS version 9.2 for Windows) software. Confirmatory factor modeling was carried out using SAS covariance analysis of linear structural equations (PROC CALIS). A two-sided *P*  value of less than  .05 was considered as statistically significant.

## 3. Results

### 3.1. Patient Characteristics

A total of 212, 159, and 133 patients at weeks 4, 8, and 12, respectively, had at least partial data. Of these patients, 159 (75%), 129 (81%), and 106 (80%) had complete data at weeks 4, 8, and 12, respectively, and this cohort formed the study population for analyses on pain and interference. Of the study population at week 4, median age was 65 (range: 30–89), 88 of patients (55%) were male, and 94 patients (60%) were classified as having lower body pain (Table 1). At week 8, median age was 66 (range: 30–88), 70 (54%) of patients were male, and 76 patients were classified as having lower body pain. Finally, at week 12, median age was 64 (range: 30–88), 62 (58%) of patients were male, and 74 patients had lower body pain. Two patients were excluded for having treatment to the thoracolumbar spine. The most commonly missed item was normal work with 36 patients missing this value at 4 weeks and 19 patients missing this score for both weeks 8 and 12.

### 3.2. Pattern of Pain, Activity, and Affect Interference in Lower versus Upper Skeletal Pain at Follow-up

Analysis of baseline data showed that patients with lower skeletal pain had significantly greater activity interference than those with upper skeletal pain; this is similar to the result obtained by Wu et al. [11] in their previous study. Analysis after treatment with conventional radiotherapy showed that there was no significant difference in pain or interference scores for lower and upper skeletal pain sites at 4 and 8 weeks (Table 2). At week 4, mean values for pain, activity, and affect in patients with lower skeletal pain were 3.87, 5.12, and 3.52, respectively, in comparison to 3.68, 4.79, and 3.96 for those with upper skeletal pain (all *P*  values not statistically significant). At week 8, mean values for pain, activity, and affect in patients with lower skeletal pain were 3.69, 4.63, and 3.39, respectively, in comparison to 3.42, 3.57, and 2.98 for those with upper skeletal pain (all *P*  values not statistically significant). At 12 weeks posttreatment, pain was significantly worse for patients with upper skeletal pain than those with lower skeletal pain (*P* = .0278), but interference levels (both activity and affect) were similar.

### 3.3. Item Analysis and Internal Consistency of Subscales

There was internal consistency among all items except sleep at each follow-up period. Improvement in correlation was seen at each follow-up period and in both two- and three-factor subscales when the sleep item was improved. Alpha for two-factor analysis improved from 0.48 to 0.91 at 4 weeks, 0.54 to 0.91 at 8 weeks, and finally, 0.56 to 0.91 at 12 weeks. For the three-factor analysis, removal of sleep from the activity subscale improved alpha from 0.45 to 0.87 at 4 weeks, 0.48 to 0.90 at 8 weeks, and 0.53 to 0.90 at 12 weeks (Table 3). In all cases, removal of sleep indicated a more reliable construct.

### 3.4. Confirmatory Factor Analysis

In general, both the two-factor and the three-factor models demonstrated reasonably high levels of internal consistency, composite reliability, and convergent validity (Tables 4 and 5). The null model (one-factor) demonstrated the worst fit of the three; this was expected given the well-established two-factor solution to the BPI. The two-factor analysis performed poorly, however, better than the null model; the three-factor model had the best performance. For the two- and three-factor analyses, considerable improvement in fit was seen by removing the sleep item and also by allowing specific error terms to covary. This result was seen at 4 weeks, 8 weeks, and 12 weeks posttreatment. 

Factor loading and associate statistics for the best two-factor and three-factor analyses were constructed at each follow-up period. The correlation between grouped items (e.g., pain, activity, and affect in three-factor analysis) was high for all three data sets (Table 6 shows week 4 as an example). At each follow-up period, there was a stronger correlation between activity and affect, which could be indicative of the same latent variable.

### 3.5. Discriminant Validity Tests for Evaluating Activity and Affect Factors

Discriminant validity tests provided mixed support for activity and affect at weeks 4, 8, and 12. Although the confidence interval (CI) test indicated correlation of 0.88, 0.87, and 0.85 at weeks 4, 8, and 12, respectively, (all within the 95% CI at each period), the Chi-square difference test demonstrated significance between mixed and separated support at all followups. The variance-extracted test failed to confirm discriminant validity for all three sets of data. Despite these results, the combination of different analyses confirmed the validity of the construct since reasonably high levels of internal consistency, composite reliability, and convergent validity were present at weeks 4, 8, and 12. 

Although the two-factor model is adequate, the three-factor model is preferred since fewer covariance terms were needed to fit the model. Furthermore, this model satisfies an additional IMMPACT recommendation.

## 4. Discussion

Functional interference is a significant component of quality of life in advanced cancer patients which has seldom been addressed in the literature. It has been shown that improvement in metastatic bone pain will similarly improve functional interference as a result of this pain [11]. The BPI is a validated and reliable tool used to assess pain and the functional interference in patients with bone metastases; however, its validity has not been confirmed in telephone use. It had previously been reported that those experiencing lower body pain have higher levels of functional interference than those with upper body pain [11]. In this analysis, we found that after treatment with conventional radiotherapy, both groups of patients have similar levels of functional interference. Further, we validate the BPI through telephone for patients with bone metastases.

The fact that interference levels are worse in lower skeletal pain patients prior to radiotherapy suggests that in general people exhibiting lower body pain will have more functional interference than those with upper body pain, not only inclusive to patients with bone metastases pain. Studies, however, have suggested that there is an inherent difference among pain interference between cancer and noncancer patients [21] meaning that even if data existed for this topic in noncancer patients, it might be inappropriate to compare the results. Overall, our findings suggest that rapid management of pain stemming from bone metastases is crucial to reduce functional interference and improve quality of life, especially in those with pain in the lower skeleton. Radiotherapy reduces functional interference as a result of pain regardless of location and shrinks the difference in functional interference between upper and lower body metastatic bone pain such that it is not significant.

The BPI has been used in numerous studies via telephone, to assess a patient's response to treatment. Although the questions remained the same whether asking a patient across the phone or in person, the differences in setting may affect responses and consequently the validity of the BPI. When comparing the statistical analyses to results of Wu et al., who validated the BPI in patients with bone metastases physically in clinic with the same methods [11], the similar high construct validity and high levels of correlation within the subscales of the BPI demonstrate its validity for use over the telephone. This modality should be recommended when following patients towards the end of life, as it reduces the burden caused by numerous clinic visits.

The sleep item of BPI contributes little to both the activity or affect subscales of three-factor analysis and interference in general in the two-factor analysis. Previous papers have also noted this difference when conducting an analysis of the BPI [11, 22–24]. The lack of correlation in these subgroups is surprising in this highly symptomatic population. Two factors contributing to this could have been due to the side effects of medications (especially the greater levels of opioids taken by cancer patients) and the patient adjusting to reduce pain interference on sleep (i.e., turning to sleep on one's side). The complex nature of the relationship between pain and sleep should be explored further.

A further point of research would be to test the correlation of the BPI with other musculoskeletal functional scales, such as the Western Ontario and McMaster University Osteoarthritis Index (WOMAC) [25] and the Musculoskeletal Function Assessment (MFA) [26] questionnaire. This could assist in confirming our findings within this population. Another limitation within our setting was the fact that the most commonly missed item of the BPI was “normal work”. The majority of people missing this item were elderly patients with decreased performance status. This population may view themselves as no longer being capable of doing normal work and feel they are not required to answer this question. As a result, these patients might not have been represented sufficiently in our population. Although expected in this population, a final limitation would be the high rates of attrition. Towards the end of the follow-up period especially, our results may reflect the patients with higher performance status. 

The BPI is a valid and reliable tool for use across the telephone and should be the preferred mode of contact when following up with advanced cancer patients. CFA of the BPI demonstrates validity with both the two- and three-factor models; however, the latter is preferred as fewer covariance terms are needed to fit the model and it satisfies an additional IMMPACT factor. Patients with lower skeletal pain resulting from bone metastases should receive prompt treatment to reduce their functional interference and improve their overall quality of life. The preferred modality for following palliative patients with quality-of-life assessments should be via the telephone as it reduces the travelling burden associated with multiple visits to a health care setting.

##  Conflict of Interests

The authors have no conflict of interests to disclose.

## Figures and Tables

**Table 1:** Patient demographics at weeks 4, 8, and 12 (with complete data sets).

Demographics	Week 4	Week 8	Week 12
Age (year)			
*n*	159	129	106
Mean ± SD	63.8 ± 13.1	63.9 ± 13.5	63.4 ± 13.8
Interquartiles	54–74	54–74	54–74
Median (Range)	65 (30–89)	66 (30–88)	64 (30–88)

Karnofsky performance status (KPS)			
*n*	151	124	104
Mean ± SD	71.9 ± 13.1	72.6 ± 11.8	72.5 ± 12.5
Interquartiles	60–80	70–80	70–80
Median (range)	70 (30–90)	70 (40–90)	70 (40–90)

Worst pain			
*n*	159	129	106
Mean ± SD	5.13 ± 2.67	4.84 ± 2.58	4.75 ± 2.44
Interquartiles	3–7	3–7	3–7
Median (range)	5.0 (1–10)	5.0 (1–10)	4.0 (1–10)

Average pain			
*n*	159	129	106
Mean ± SD	3.57 ± 2.28	3.60 ± 2.20	3.33 ± 2.12
Interquartiles	2–5	2–5	2–5
Median (range)	3.0 (0–10)	3.0 (0–9)	3.0 (0–9)

Current pain			
*n*	159	129	106
Mean ± SD	2.69 ± 2.49	2.33 ± 2.26	2.41 ± 2.37
Interquartiles	0–4	0–4	0–4
Median (range)	3.0 (0–10)	2.0 (0–10)	2.0 (0–9)

Total daily morphine equivalent (mg)			
*n*	143	115	94
Mean ± SD	101.4 ± 162.5	91.6 ± 133.2	119.6 ± 194.5
Interquartiles	0–135	0–135	0–210
Median (range)	30 (0–904)	32 (0–832)	24 (0–1080)

Gender			
Male	88 (55.35%)	70 (54.26%)	62 (58.49%)
Female	71 (44.65%)	59 (45.74%)	44 (41.51%)

Pain site			
Lower limb	94 (59.12%)	76 (58.91%)	74 (69.81%)
Upper limb	63 (39.62%)	50 (38.76%)	29 (27.36%)
Other	2 (1.26%)	3 (2.33%)	3 (2.83%)

Primary cancer site			
Prostate	46 (28.93%)	41 (31.78%)	32 (30.19%)
Breast	37 (23.27%)	31 (24.03%)	28 (26.42%)
Lung	41 (25.79%)	28 (21.71%)	24 (22.64%)
Bladder	10 (6.29%)	8 (6.20%)	6 (5.66%)
Pancreas/gastric	5 (3.14%)	0 (0.00%)	1 (0.94%)
Others	20 (12.58%)	37 (17.5%)	15 (14.15%)

Dose fractionation			
Single	100 (62.89%)	72 (55.81%)	67 (63.21%)
Multiple	59 (37.11%)	57 (44.19%)	39 (36.79%)

**Table 2:** One-way ANOVA comparing upper versus lower skeletal pain at 4, 8, and 12 weeks posttreatment.

Subscales	Lower skeletal pain		Upper skeletal pain			Mean difference (95% CI)
	Mean	SD	Mean	SD	*P* value	
At week 4	*n* = 94		*n* = 63			
Mean pain	3.87	2.25	3.68	2.23	.6110	0.19 (−0.54–0.91)
Mean activity	5.12	3.22	4.79	3.24	.5347	0.33 (−0.71–1.36)
Mean affect	3.52	2.79	3.96	3.24	.3645	0.44 (−0.52–1.40)

At week 8	*n* = 76		*n* = 50			
Mean pain	3.69	2.13	3.42	1.96	.4765	0.27 (−0.48–1.01)
Mean activity	4.63	3.37	3.57	2.92	.0730	1.05 (−0.10–2.21)
Mean affect	3.39	2.77	2.98	2.66	.4153	0.41 (−0.58–1.39)

At week 12	*n* = 74		*n* = 29			
*Mean pain*	**3.22**	**1.93**	**4.23**	**2.37**	**.0278**	**1.01 (0.11–1.91)**
Mean activity	4.22	3.09	4.45	3.45	.7460	0.23 (−1.16–1.62)
Mean affect	2.58	2.33	2.99	2.73	.4437	0.41 (−0.65–1.47)

There is no significant difference in functional interference (both activity and affect—three-factor analysis) between upper and lower skeletal pain groups after radiation. Mean pain is significantly greater at 12 weeks for the upper skeletal pain group (*P* = .0278).

**Table 3:** BPI internal consistency using Cronbach's alpha.

			Standardized Cronbach's alpha			
	Item statistics		Two subscales		Three subscales	
BPI items	Mean	SD	Correlation with total	Alpha with item deleted	Correlation with total	Alpha with item deleted
At week 4		
			Pain subscale (alpha = 0.88)		Pain subscale (alpha = 0.88)	
Worst pain	5.1	2.7	0.75	0.85	0.75	0.85
Average pain	3.6	2.3	0.83	0.78	0.83	0.78
Current pain	2.7	2.5	0.74	0.86	0.74	0.86
			Interference subscale (alpha = 0.90)		Activity subscale (alpha = 0.83)	
General activity	5.1	3.4	0.79	0.87	0.78	0.72
Walking ability	4.5	3.5	0.71	0.88	0.66	0.78
Normal work	5.6	3.9	0.75	0.88	0.74	0.74
*Sleep*	3.2	3.4	0.48	** 0.91 ↑**	0.45	** 0.87 ↑**
					Affect subscale (alpha = 0.84)	
Mood	3.7	3.6	0.72	0.88	0.77	0.72
Enjoyment of life	5.0	3.4	0.79	0.87	0.66	0.83
Relations	2.5	3.3	0.67	0.89	0.70	0.79

At week 8		
			Pain subscale (alpha = 0.86)		Pain subscale (alpha = 0.86)	
Worst pain	4.8	2.6	0.75	0.78	0.75	0.78
Average pain	3.6	2.2	0.84	0.70	0.84	0.70
Current pain	2.3	2.3	0.62	0.91	0.62	0.91
			Interference subscale (alpha = 0.91)		Activity subscale (alpha = 0.85)	
General activity	4.4	3.3	0.78	0.88	0.77	0.78
Walking ability	4.0	3.6	0.75	0.89	0.75	0.79
Normal work	4.5	3.8	0.80	0.88	0.79	0.77
*Sleep*	2.5	2.8	0.54	** 0.91 ↑**	0.48	** 0.90 ↑**
					Affect subscale (alpha = 0.83)	
Mood	3.4	3.2	0.80	0.88	0.76	0.71
Enjoyment of life	4.2	3.3	0.73	0.89	0.67	0.80
Relations	2.3	3.1	0.64	0.90	0.67	0.80

At week 12		
			Pain subscale (alpha = 0.88)		Pain subscale (alpha = 0.88)	
Worst pain	4.7	2.4	0.76	0.85	0.76	0.85
Average pain	3.3	2.1	0.85	0.76	0.85	0.76
Current pain	2.4	2.4	0.71	0.88	0.71	0.88
			Interference subscale (alpha = 0.90)		Activity subscale (alpha = 0.86)	
General activity	4.5	3.3	0.80	0.88	0.78	0.80
Walking ability	3.9	3.5	0.77	0.88	0.77	0.80
Normal work	4.4	3.8	0.78	0.88	0.79	0.80
*Sleep*	2.8	3.2	0.56	** 0.91 ↑**	0.53	** 0.90 ↑**
					Affect subscale (alpha = 0.82)	
Mood	2.9	2.8	0.70	0.89	0.69	0.72
Enjoyment of life	3.4	3.0	0.77	0.88	0.72	0.70
Relations	2.0	2.9	0.64	0.90	0.60	0.82

A marked improvement was seen across all follow-up periods when the sleep item was removed from the BPI analysis.

**Table 4:** Model-fit statistics for two-factor analysis and improvement after sleep removal at weeks 4, 8, and 12.

Model	Modification	Goodness of fit index (adjusted)	Chi-square (df)	RMSEA (upper CL)	Comparative fit index	Nonnormed fit index
Model fit statistics at week 4						
2 factors: pain and interference	Covary error terms of average-pain-current pain, mood-relations, mood-enjoyment, relations-enjoyment	0.95	47.1 (28)	0.07 (0.10)	0.98	0.97
2 factors: pain and interference	Drop sleep: Covary error terms of average-pain-current pain, mood-relations, mood-enjoyment, relations-enjoyment	**0.97**	**26.3 (20)**	**0.04 (0.09)**	**0.99**	**0.99**

Model fit statistics at week 8						
2 factors: pain and interference	Covary error terms of average-pain-current pain, mood-relations, mood-enjoyment, relations-enjoyment	0.95	34.5 (28)	0.04 (0.08)	0.99	0.99
2 factors: pain and interference	Drop sleep; covary error terms of average pain-current pain, mood-relations, mood-enjoyment, relations-enjoyment	**0.96**	**25.2 (20)**	**0.05 (0.09)**	**0.99**	**0.99**

Model fit statistics at week 12						
2 factors: pain and interference	Covary error terms of average-pain-current pain, mood-relations, mood-enjoyment, relations-enjoyment	0.92	46.2 (28)	0.08 (0.12)	0.97	0.96
2 factors: pain and interference	Drop sleep; covary error terms of average-pain-current pain, mood-relations, mood-enjoyment, relations-enjoyment	**0.94**	**33.3 (20)**	**0.08 (0.13)**	**0.98**	**0.96**

Acceptable model fit		Perfect fit = 1	Smaller value	RMSEA < 0.10	**>0.90**	**>0.90**

Model fit statistics were all improved after dropping sleep, at all time periods for two-factor analysis.

**Table 5:** Model-fit statistics for three-factor analysis and improvement after sleep removal at weeks 4, 8, and 12.

Model	Modification	Goodness of fit index (adjusted)	Chi-square (df)	RMSEA (upper CL)	Comparative fit index	Nonnormed fit index
Model fit statistics at week 4						
3 factors: pain, activity and affect	Covary error terms of average-pain-current pain, mood-relations	0.95	46.8 (27)	0.07 (0.10)	0.98	0.97
3 factors: pain, activity and affect	Drop sleep: covary error terms of average pain-current pain, mood-relations	**0.98**	**26.1 (19)**	**0.05 (0.09)**	**0.99**	**0.99**

Model fit statistics at week 8						
3 factors: pain, activity and affect	Covary error terms of average-pain-current pain, mood-relations	0.95	35.6 (27)	0.05 (0.09)	0.99	0.98
3 factors: pain, activity and affect	Drop sleep; covary error terms of average-pain-current pain, mood-relations	**0.96**	**26.8 (19)**	**0.06 (0.10)**	**0.99**	**0.98**

Model fit statistics at week 12						
3 factors: pain, activity and affect	Covary error terms of average-pain-current pain, mood-relations	0.92	46.7 (27)	0.08 (0.12)	0.97	0.95
3 factors: pain, activity and affect	Drop sleep; covary error terms of average-pain-current pain, mood-relations	**0.94**	**33.5 (19)**	**0.08 (0.13)**	**0.98**	**0.96**

Acceptable model fit		Perfect fit = 1	Smaller value	RMSEA < 0.10	**>0.90**	**>0.90**

Similarly, model fit statistics were all improved after dropping sleep, at all time periods for two-factor analysis.

**Table 6:** Week 4 factor loading and associate statistic analysis.

BPI items	Two subscales			Three subscales		
	Factor loading	*R*-squared	*t*-statistic	Factor loading	*R*-squared	*t*-statistic
	Composite reliability pain factor 0.85			Composite reliability pain factor 0.82		
Worst pain	0.83	0.73	17.2	0.81	0.73	16.9
Average pain	0.86	0.81	19.2	0.82	0.81	18.7
Current pain	0.73	0.62	14.8	0.71	0.62	14.5
	Composite reliability interference factor 0.86			Composite reliability activity factor 0.81		
General activity	0.79	0.72	18.5	0.82	0.76	18.0
Walking ability	0.71	0.61	15.1	0.72	0.62	14.5
Normal work	0.72	0.65	16.2	0.75	0.69	16.1
				Composite reliability affect factor 0.76		
Mood	0.70	0.60	14.7	0.75	0.70	16.7
Enjoyment of life	0.70	0.65	16.3	0.73	0.66	15.8
Relations	0.65	0.48	11.8	0.68	0.57	13.6

	Correlation pain-interference: 0.72			Correlation pain-activity: 0.73		
				Correlation pain-affect: 0.67		
				Correlation activity-affect: 0.88		

It is shown here that the correlation between the subsections of the BPI (in both two- and three- factor analyses) is relatively high. This same result is found in week 8 and week 12. In all cases, activity and affect demonstrate a stronger correlation, suggesting the same latent variable.
